# Association of ideal body weight–normalized post-transplantation cyclophosphamide dose with chronic GVHD in patients with overweight/obesity

**DOI:** 10.1038/s41409-026-02928-8

**Published:** 2026-06-20

**Authors:** Tomohiro Nagano, Takumi Kondo, Yusuke Inoue, Aya Komura, Ryuichiro Hiyama, Keisuke Seike, Shuntaro Ikegawa, Hideaki Fujiwara, Noboru Asada, Daisuke Ennishi, Keiko Fujii, Nobuharu Fujii, Yoshinobu Maeda

**Affiliations:** 1 Department of Hematology, Oncology and Respiratory Medicine, Dentistry and Pharmaceutical Sciences, Okayama University Graduate School of Medicine, Okayama, Japan; 2 Department of Hematology, Okayama University Hospital, Okayama, Japan; 3 Department of Medical Oncology, Okayama University Hospital, Okayama, Japan; 4 Division of Clinical Laboratory, Okayama University Hospital, Okayama, Japan; 5 Division of Transfusion and Cell Therapy, Okayama University Hospital, Okayama, Japan

**Keywords:** Haematological cancer, Graft-versus-host disease

## Introduction

Post-transplantation cyclophosphamide (PTCy) is widely used for graft-versus-host disease (GVHD) prophylaxis in haploidentical hematopoietic stem cell transplantation. Although the standard dose is 100 mg/kg over 2 days, reduced-dose schedules with preserved GVHD control have been explored [1–5].

PTCy dosing uses different body-weight metrics. Recent pharmacokinetic analyses suggest ideal body weight (IBW)-based dosing better reflects cyclophosphamide metabolite exposure than total body weight (TBW)-based dosing [5]. However, overweight/obese recipients have rarely been evaluated in prior PTCy reports [6], and the role of body size remains unclear. Therefore, we evaluated the association between IBW- and TBW-based PTCy dosing and chronic GVHD (cGVHD) in overweight and obese transplant recipients.

## Subjects and methods

We retrospectively analyzed adults (≥18 years) who underwent haploidentical peripheral blood stem cell transplantation (Haplo-PBSCT) with PTCy at Okayama University Hospital between April 2017 and December 2024.

Overweight and obesity were defined as body mass index (BMI) 25.0–29.9 and ≥30.0 kg/m², respectively [7], and non-overweight as BMI < 25.0 kg/m². Cyclophosphamide dosing was based on the actual administered dose rather than planned weight-based doses. The total dose over the two PTCy days was recalculated as TBW- and IBW-normalized doses, with IBW calculated using the Devine method [8]. Adjusted body weight (ABW25) was explored, as it incorporates both TBW and IBW.

As severe acute GVHD (aGVHD) events were infrequent, cumulative cGVHD incidence was the primary outcome. Exploratory outcomes included aGVHD, neutrophil and platelet engraftment, overall survival, relapse, non-relapse mortality, complications, and infections. Cumulative incidence was estimated using the cumulative incidence function and compared with Gray’s test, treating relapse, subsequent transplantation, and death as competing events. Given the small sample size and limited follow-up, exploratory receiver operating characteristic (ROC) analyses were performed to derive binary cGVHD dose cutoffs within 1 year. As multivariable analysis was not feasible, potential confounding was assessed through stratified analyses. Outcomes in non-overweight recipients were also explored. Additional definitions and statistical methods are provided in the Supplementary Methods.

This study was approved by the Ethics Committee of Okayama University Hospital (No. 2508-024) and conducted in accordance with the Declaration of Helsinki and the Ethical Guidelines for Medical and Biological Research Involving Human Subjects in Japan. Because of its retrospective design using routinely collected clinical information, the requirement for written informed consent was waived by the Ethics Committee, and all patients were offered the opportunity to opt out.

## Results

Ninety-four adult patients underwent Haplo-PBSCT with PTCy (12 were overweight, and five were obese). The characteristics of overweight and obese recipients are presented in Supplementary Table 1. PTCy dosing was planned using either IBW or ABW25, and the median administered doses were 78.7 (TBW-normalized) and 101.5 mg/kg (IBW-normalized) (Supplementary Fig. 1a). GVHD prophylaxis included tacrolimus and mycophenolate mofetil in addition to PTCy. Tacrolimus was initiated after PTCy in 10 patients, and before PTCy in seven patients according to physician’s discretion based on disease status and transplantation setting.

**Fig. 1:**
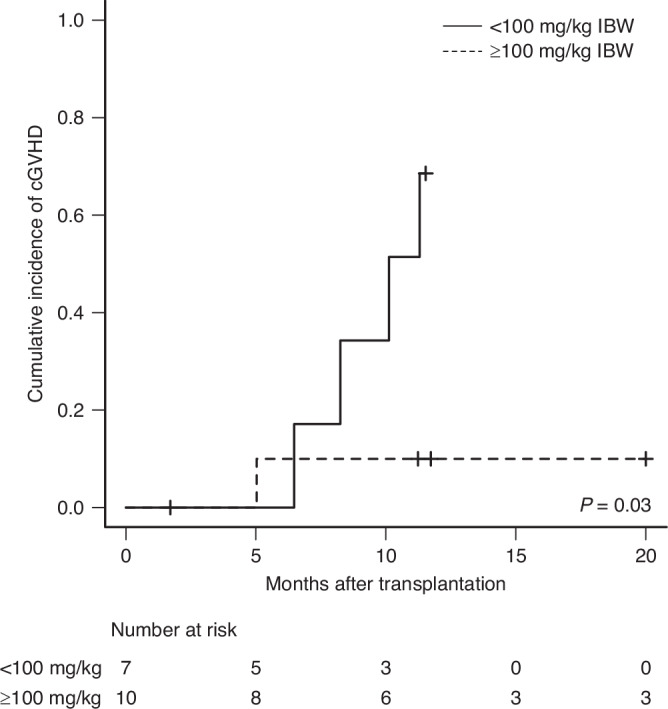
Cumulative incidence of chronic GVHD according to IBW-normalized total PTCy dose in overweight and obese recipients. The cumulative incidence of chronic graft-versus-host disease (cGVHD) was compared between overweight/obese recipients who received <100 mg/kg ideal body weight (IBW) and ≥100 mg/kg IBW total post-transplantation cyclophosphamide (PTCy). The 1-year cumulative incidence was 68.5% (95% CI, 21.3–91.2) in the <100 mg/kg IBW group, and 10.0% (95% CI, 0.6–35.8) in the ≥100 mg/kg IBW group (*p* = 0.03). Cumulative incidence curves were estimated using the cumulative incidence function, treating relapse, subsequent transplantation, and death as competing events. Group comparisons were performed using Gray’s test. Tick marks indicate censoring, and numbers at risk are presented below the plot.

Exploratory ROC analyses identified rounded cutoffs of 100 mg/kg (IBW-normalized) and 75 mg/kg (TBW-normalized) (Supplementary Fig. 1b, c). The cumulative cGVHD incidence was higher among patients receiving <100 than among those receiving ≥100 mg/kg IBW (68.5% [95% confidence interval {CI}, 21.3–91.2] vs. 10.0% [95% CI, 0.6–35.8] at 1 year, *p* = 0.03; Fig. 1), whereas the difference between <75 and ≥75 mg/kg TBW was not statistically significant (Supplementary Fig. 1d). ABW25-based stratification showed a similar, although non-significant pattern to IBW-based stratification (Supplementary Fig. 1e, f). These results should be interpreted cautiously, given the small sample size and wide confidence intervals.

Patient characteristics differed between those receiving ≥100 and <100 mg/kg IBW (Supplementary Table 1). However, in the stratified analyses, the direction of the association between PTCy dose and cumulative cGVHD incidence did not clearly reverse, although subgroup sizes were small (Supplementary Table 2).

Patients treated with <100 mg/kg IBW showed a trend toward higher incidences of moderate-to-severe cGVHD and aGVHD than those who received ≥100 mg/kg IBW; however, these differences were not significant. No patients in our cohort developed grade-3–4 aGVHD. Platelet engraftment occurred later in patients who received ≥100 mg/kg IBW compared with those who received <100 mg/kg IBW. Neutrophil engraftment was comparable between the groups (Supplementary Fig. 2a–d). The cumulative incidences of relapse and non-relapse mortality and overall survival were comparable between the dose groups (Supplementary Fig. 3a–c).

Complications and infections, including cytomegalovirus-related events, hemorrhagic cystitis, and cardiac events, did not differ significantly between patients who received ≥100 or <100 mg/kg IBW, although the analyses were limited by sample size (Supplementary Table 3).

Among non-overweight recipients, using the same 100 mg/kg IBW cutoff, cumulative cGVHD incidence did not differ between dose groups (Supplementary Table 4 and Supplementary Fig. 4a–c). Among recipients receiving <100 mg/kg IBW, overweight and obese individuals exhibited higher incidence of cGVHD than non-overweight recipients, whereas no such difference was observed among those receiving ≥100 mg/kg IBW.

## Discussion

In our study, lower IBW-normalized PTCy was associated with higher cGVHD incidence among recipients with BMI ≥ 25 kg/m², but not among those with BMI < 25 kg/m², suggesting that the effect of reduced-dose PTCy may vary by body size. Because prior PTCy studies have rarely incorporated body-size measures, this issue remains underexplored.

Pharmacometric analyses of high-dose cyclophosphamide suggest that IBW- or ABW25-normalized dosing may reduce predicted metabolite exposure as BMI increases [9], offering a biologically plausible explanation. However, the link between cyclophosphamide metabolite exposure and cGVHD remains unclear. One Haplo-PBSCT study reported an association with severe cGVHD [10], whereas a haploidentical bone marrow transplantation study did not, although metabolite exposure correlated with several clinical endpoints [5].

Although several studies have shown acceptable GVHD control with reduced-dose PTCy, these findings do not necessarily conflict with ours, as recipient body size has rarely been analyzed. Interpretation of cGVHD outcomes in prior studies is limited by heterogeneous reporting and comparator groups [1], and cGVHD has seldom been prospectively assessed as a primary endpoint [5]. Our study is distinct in evaluating cGVHD in overweight and obese recipients, an approach rarely taken in reduced-dose PTCy reports.

Engraftment kinetics, particularly platelet engraftment, showed modest delays consistent with prior reports [2–5], although interpretation is limited by imbalance in CD34^+^ cell doses. No clear differences in complications were observed.

This study has several limitations. First, as a single-center retrospective study with a small number of overweight or obese recipients, risks of type I error for GVHD outcomes and type II error for complications and analyses in the non-overweight group cannot be excluded. The small sample size precluded robust multivariable analysis, and the stratified analyses were limited by small subgroup sizes, making it difficult to exclude residual confounding from baseline imbalances. Second, the lack of pharmacokinetic data limited mechanistic interpretation. Third, the ROC–derived cutoffs should be considered exploratory, as conventional methods do not account for time-to-event outcomes, censoring, or competing risks. Finally, part of the cohort overlapped with our prior report on tacrolimus initiation timing [11]. Although tacrolimus initiation timing was similar across IBW-defined dose groups, residual confounding cannot be excluded. Therefore, these findings should be considered hypothesis-generating rather than causal.

In summary, our findings suggest that body size may influence the clinical interpretation of reduced-dose PTCy strategies in overweight and obese recipients. Given the small sample size and potential confounding, these observations should be considered hypothesis-generating and require validation in multicenter studies incorporating pharmacokinetic analyses.

## Supplementary information

Supplementary Materials

## Data Availability

The datasets generated during and/or analyzed during the current study are available from the corresponding author on reasonable request.
