# Supplementary material for: Association of ideal body weight–normalized post-transplantation cyclophosphamide dose with chronic GVHD in patients with overweight/obesity
Source: Bone Marrow Transplant. 2026 Jun 20;61(8):1118–20. doi: 10.1038/s41409-026-02928-8 (PMC13437231; doi:10.1038/s41409-026-02928-8)
Supplement: Supplementary file 1 [file 41409_2026_2928_MOESM1_ESM.pdf]

Supplementary Table 1.

Baseline and transplant characteristics of overweight/obese recipients stratified by IBW-normalized total PTCy dose.

		All Patients	<100 mg/kg IBW	≥100 mg/kg IBW	p value
n		17	7	10	
Follow-up period (days)		365 [39 - 1497]	365 [39 - 1182]	404.5 [77 - 1497]	0.77
Age		50 [25 - 72]	54 [38 - 72]	45 [25 - 60]	0.09
Sex	Male	14 (82.4%)	7 (100.0%)	7 (70.0%)	0.23
	Female	3 (17.6%)	0 (0.0%)	3 (30.0%)	
Height (cm)		168.6 [157.6 - 180.2]	168.0 [158.0 - 175.7]	170.6 [157.6 - 180.2]	0.50
Body weight (kg)		77.9 [66.3 - 110.0]	76.0 [66.3 - 80.4]	83.65 [72.8 - 110.0]	0.05
BMI		27.1 [25.0 - 36.7]	26.6 [25.3 - 30.6]	29.3 [25.0 - 36.7]	0.04
CCr (mL/min)		81.4 [30.8 - 170.0]	81.4 [52.6 - 105.8]	93.8 [30.8 - 170.0]	0.33
Disease	AML	5 (29.4%)	4 (57.1%)	1 (10.0%)	0.32
	MDS	1 (5.9%)	0 (0.0%)	1 (10.0%)	
	CML	2 (11.8%)	1 (14.3%)	1 (10.0%)	
	MPN not CML	2 (11.8%)	0 (0.0%)	2 (20.0%)	
	ALL	4 (23.5%)	2 (28.6%)	2 (20.0%)	
	ML	2 (11.8%)	0 (0.0%)	2 (20.0%)	
	ATLL	1 (5.9%)	0 (0.0%)	1 (10.0%)	
Conditioning intensity	MAC	8 (47.1%)	2 (28.6%)	6 (60.0%)	0.34
	RIC	9 (52.9%)	5 (71.4%)	4 (40.0%)	
Conditioning regimen	FLU/BU	9 (52.9%)	4 (57.1%)	5 (50.0%)	1.00
	FLU/MEL	7 (41.2%)	3 (42.9%)	4 (40.0%)	
	FLU/TBI	1 (5.9%)	0 (0.0%)	1 (10.0%)	
TBI	2 Gy	4 (23.5%)	2 (28.6%)	2 (20.0%)	1.00
	4 Gy	12 (70.6%)	5 (71.4%)	7 (70.0%)	
	12 Gy	1 (5.9%)	0 (0.0%)	1 (10.0%)	
Sex mismatch	M to M	8 (47.1%)	5 (71.4%)	3 (30.0%)	0.45
	F to M	6 (35.3%)	2 (28.6%)	4 (40.0%)	
	F to F	2 (11.8%)	0 (0.0%)	2 (20.0%)	
	M to F	1 (5.9%)	0 (0.0%)	1 (10.0%)	
Tacrolimus initiation	Before PTCy	7 (41.2%)	3 (42.9%)	4 (40.0%)	1.00
	After PTCy	10 (58.8%)	4 (57.1%)	6 (60.0%)	
CD34+ cells ($\times 10^6$ /kg)		4.01 [2.61 - 7.30]	5.27 [3.52 - 7.30]	3.62 [2.61 - 6.45]	0.04

Categorical variables are presented as n (%), and continuous variables are presented as the median with minimum and maximum values. P values were calculated for comparisons between the <100 mg/kg IBW and ≥100 mg/kg IBW total PTCy groups using Fisher's exact test for categorical variables and the Wilcoxon rank-sum test for continuous variables.

Abbreviations: ALL, acute lymphoblastic leukemia; AML, acute myeloid leukemia; ATLL, adult T-cell leukemia/lymphoma; BMI, body mass index; BU, busulfan; CCr, creatinine clearance; CML, chronic myeloid leukemia; F, Female; FLU, fludarabine; HSCT, hematopoietic stem cell transplantation; IBW, ideal body weight; M, Male; MAC, myeloablative conditioning; MDS, myelodysplastic syndrome; MEL, melphalan; ML, malignant lymphoma; MPN, myeloproliferative neoplasm; PTCy, post-transplant cyclophosphamide; RIC, reduced-intensity conditioning; TBI, total body irradiation.

Supplementary Table 2.

Stratified (subgroup) analyses of 1-year cumulative incidence of cGVHD by IBW-normalized total PTCy dose in overweight/obese recipients.

		1-year cumulative incidence of cGVHD (%)		
		<100 mg/kg IBW	≥100 mg/kg IBW	p value
Age	<60 years old	50.0 (5.8 - 84.5)	11.1 (0.6 - 38.8)	0.19
	≥60 years old	100.0 (NA)	0.0 (NA)	0.23
Sex	Male	68.6 (21.3 - 91.2)	0.0 (NA)	0.01
Overweight/Obese	Overweight	62.5 (14.2 - 89.3)	0.0 (NA)	0.03
	Obese	100.0 (NA)	25.0 (0.9 - 66.5)	0.35
CCr	<81.4 mL/min	66.7 (5.4 - 94.5)	20.0 (0.8 - 58.2)	0.35
	≥81.4 mL/min	66.7 (5.4 - 94.5)	0.0 (NA)	0.05
Conditioning intensity	RIC	80 (20.4 - 96.9)	0.0 (NA)	0.04
	MAC	50.0 (0.6 - 91.0)	16.7 (0.8 - 51.7)	0.46
Conditioning regimen	FLU/BU	75.0 (12.8 - 96.1)	20.0 (0.8 - 58.2)	0.23
	FLU/MEL	66.7 (5.4 - 94.5)	0.0 (NA)	0.12
Sex mismatch	M to M	53.3 (6.8 - 86.3)	0.0 (NA)	0.21
	F to M	100.0 (NA)	0.0 (NA)	0.03
Timing of tacrolimus initiation	Before PTCy	66.7 (5.4 - 94.5)	25.0 (0.9 - 66.5)	0.55
	After PTCy	75.0 (12.8 - 96.1)	0.0 (NA)	0.02
Received CD34+ cells	$<4.01 \times 10^6$ /kg	50.0 (0.6 - 91.0)	0.0 (NA)	0.12
	$\geq 4.01 \times 10^6$ /kg	75.0 (12.8 - 96.1)	25.0 (0.9 - 66.5)	0.30

Values are 1-year cumulative incidence (%) with 95% confidence intervals from the cumulative incidence function (competing events: relapse, subsequent transplantation, death); P values are from Gray's test. Creatinine clearance and received CD34+ cell dose were dichotomized at the median; analyses were exploratory and unadjusted for multiple comparisons.

Abbreviations: NA, not applicable; other abbreviations as in Supplementary Table 1.

Supplementary Table 3.

Major complications and infections in overweight/obese recipients stratified by IBW-normalized total PTCy dose.

	<100 mg/kg IBW	≥100 mg/kg IBW	p value
CMV infection	0/7 (0.0%)	0/10 (0.0%)	NA
CMV reactivation	1/7 (14.3%)	4/10 (40.0%)	0.34
BKV-associated hemorrhagic cystitis	1/7 (14.3%)	2/10 (20.0%)	1.00
Hemorrhagic cystitis without virus detection	0/7 (0.0%)	1/10 (10.0%)	1.00
VOD/SOS	0/7 (0.0%)	2/10 (20.0%)	0.49
Arrhythmia	1/7 (14.3%)	0/10 (0.0%)	0.41
Acute heart failure	0/7 (0.0%)	0/10 (0.0%)	NA
LVEF decline	0/4 (0.0%)	0/9 (0.0%)	NA
New-onset pericardial effusion	2/6 (33.3%)	2/10 (20.0%)	0.60

Values are shown as n/N (%), where n indicates the number of patients with the event and N indicates the number of evaluable patients in each group. For most outcomes, N corresponds to the total number of patients in each group. For LVEF decline, N corresponds to the number of patients with evaluable follow-up echocardiography. For new-onset pericardial effusion, N corresponds to the number of patients with evaluable post-transplant CT imaging and no pericardial effusion on pre-transplant CT. P values were calculated using Fisher's exact test.

Abbreviations: BKV, BK virus; CMV, cytomegalovirus; LVEF, left ventricular ejection fraction; NA, not applicable; VOD/SOS, veno-occlusive disease/sinusoidal obstruction syndrome; other abbreviations as in Supplementary Table 1.

Supplementary Table 4.

Baseline and transplant characteristics of non-overweight recipients stratified by IBW-normalized total PTCy dose.

		All Patients	<100 mg/kg IBW	≥100 mg/kg IBW	p value
n		77	61	16	
Follow-up period (days)		748 [31 - 2946]	735 [31 - 2946]	1051 [99 - 1957]	0.23
Age		57 [18 - 70]	57 [19 - 70]	54 [18 - 68]	0.50
Sex	Male	54 (70.1%)	47 (77.0%)	7 (43.8%)	0.02
	Female	23 (29.9%)	14 (23.0%)	9 (56.2%)	
Height (cm)		168.7 [146.9 - 181.5]	169.3 [148.5 - 181.5]	160.4 [146.9 - 175.0]	0.002
Body weight (kg)		58.8 [34.6 - 81.0]	58.8 [34.6 - 81.0]	56.6 [40.0 - 73.0]	0.91
BMI		21.1 [14.5 - 24.7]	20.6 [14.5 - 24.7]	23.0 [17.8 - 24.6]	0.01
CCr (mL/min)		90.4 [34.1 - 169.4]	89.9 [34.1 - 169.4]	98.1 [53.8 - 140.7]	0.27
Disease	AML	34 (44.2%)	27 (44.3%)	7 (43.8%)	0.31
	MDS	13 (16.9%)	12 (19.7%)	1 (6.2%)	
	CML	1 (1.3%)	1 (1.6%)	0 (0.0%)	
	MPN not CML	4 (5.2%)	4 (6.6%)	0 (0.0%)	
	ALL	12 (15.6%)	8 (13.1%)	4 (25.0%)	
	ML	9 (11.7%)	6 (9.8%)	3 (18.8%)	
	ATLL	3 (3.9%)	3 (4.9%)	0 (0.0%)	
	AA	1 (1.3%)	0 (0.0%)	1 (6.2%)	
Conditioning intensity	MAC	34 (44.2%)	27 (44.3%)	7 (43.8%)	1.00
	RIC	43 (55.8%)	34 (55.7%)	9 (56.2%)	
Conditioning regimen	FLU/BU	49 (63.6%)	38 (62.3%)	11 (68.8%)	1.00
	FLU/MEL	21 (27.3%)	17 (27.9%)	4 (25.0%)	
	FLU/TBI	7 (9.1%)	6 (9.8%)	1 (6.2%)	
TBI	2 Gy	19 (24.7%)	16 (26.2%)	3 (18.8%)	0.91
	4 Gy	51 (66.2%)	39 (63.9%)	12 (75.0%)	
	12 Gy	7 (9.1%)	6 (9.8%)	1 (6.2%)	
Sex mismatch	M to M	36 (46.8%)	29 (47.5%)	7 (43.8%)	0.01
	F to M	18 (23.4%)	18 (29.5%)	0 (0.0%)	
	F to F	8 (10.4%)	5 (8.2%)	3 (18.8%)	
	M to F	15 (19.5%)	9 (14.8%)	6 (37.5%)	
Tacrolimus initiation	Before PTCy	23 (29.9%)	21 (34.4%)	2 (12.5%)	0.13
	After PTCy	54 (70.1%)	40 (65.6%)	14 (87.5%)	
CD34+ cells (× 10^6 /kg)		5.40 [1.46 - 10.70]	5.47 [1.46 - 10.70]	5.30 [3.08 - 8.44]	0.71

Categorical variables are presented as n (%), and continuous variables are presented as the median with minimum and maximum values. P values were calculated for comparisons between the <100 mg/kg IBW and ≥100 mg/kg IBW total PTCy groups using Fisher's exact test for categorical variables and the Wilcoxon rank-sum test for continuous variables.

Abbreviations: AA, aplastic anemia; other abbreviations as in Supplementary Table 1.

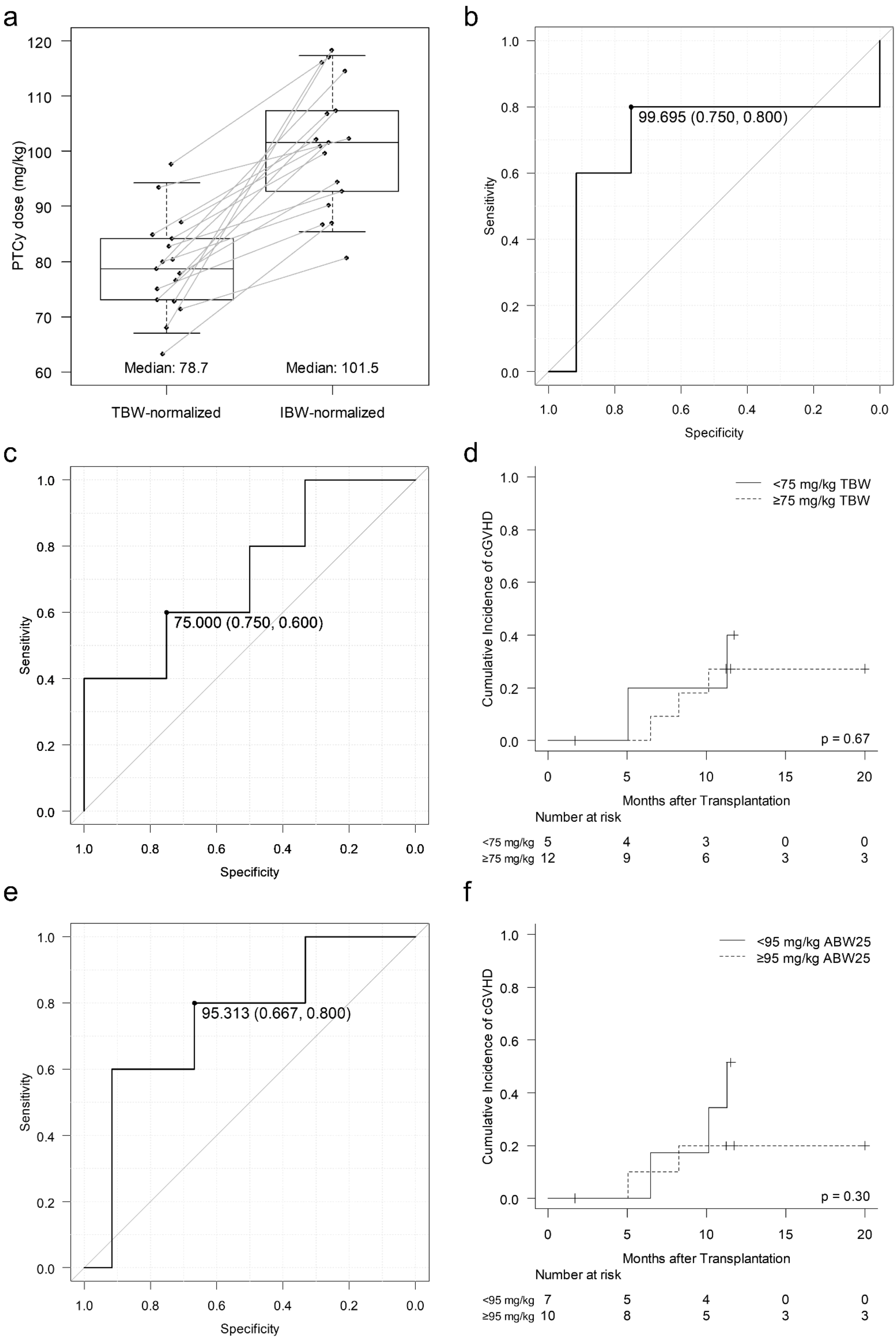
a
PTCy dose (mg/kg)
120
110
100
90
80
70
60
Median: 78.7
Median: 101.5
TBW-normalized
IBW-normalized
b
Sensitivity
Specificity
99.695 (0.750, 0.800)
1.0
0.8
0.6
0.4
0.2
0.0
c
Sensitivity
Specificity
75.000 (0.750, 0.600)
d
Cumulative Incidence of cGVHD
<75 mg/kg TBW
≥75 mg/kg TBW
p = 0.67
Months after Transplantation
0
5
10
15
20
Number at risk
<75 mg/kg 5 4 3 0 0
≥75 mg/kg 12 9 6 3 3
e
Sensitivity
Specificity
95.313 (0.667, 0.800)
f
Cumulative Incidence of cGVHD
<95 mg/kg ABW25
≥95 mg/kg ABW25
p = 0.30
Months after Transplantation
Number at risk
<95 mg/kg 7 5 4 0 0
≥95 mg/kg 10 8 5 3 3

Supplementary Figure 1. Weight-normalized total PTCy dose metrics and cumulative incidence of chronic GVHD in recipients with overweight/obesity.

(a) Distribution of total post-transplant cyclophosphamide (PTCy) dose normalized to total body weight (TBW) or ideal body weight (IBW) in the study cohort. Boxes represent the interquartile range with the median; whiskers indicate the 5th and 95th percentiles; dots represent individual recipients; lines connect paired TBW- and IBW-normalized values for each recipient. Median values are annotated in the panel.

(b) Conventional (non–time-dependent) ROC curve for IBW-normalized total PTCy dose to classify binary chronic GVHD (occurrence within 1 year). The annotated point indicates the selected cutoff.

(c) Conventional ROC curve for TBW-normalized total PTCy dose. The annotated point indicates the selected cutoff.

(d) Cumulative incidence of chronic GVHD stratified by TBW-normalized total PTCy dose (<75 mg/kg TBW vs ≥75 mg/kg TBW). The 1-year cumulative incidence was 40.0% (95% CI, 5.2–75.3) in the <75 mg/kg TBW group and 27.3% (95% CI, 6.5–53.9) in the ≥75 mg/kg TBW group (p = 0.67). Cumulative incidence was estimated using the cumulative incidence function, treating relapse, subsequent transplantation, and death as competing events; groups were compared using Gray's test. Tick marks indicate censoring; numbers at risk are shown.

(e) Conventional ROC curve for ABW25-normalized total PTCy dose. The annotated point indicates the derived cutoff.

(f) Cumulative incidence of chronic GVHD stratified by ABW25-normalized total PTCy dose (<95 mg/kg ABW25 vs ≥95 mg/kg ABW25). The 1-year cumulative incidence was 51.4% (95% CI, 11.8–81.3) in the <95 mg/kg ABW25 group and 20.0% (95% CI, 3.1–47.5) in the ≥95 mg/kg ABW25 group (p = 0.30). Cumulative incidence was estimated and compared as described in (d). Tick marks indicate censoring; numbers at risk are shown.

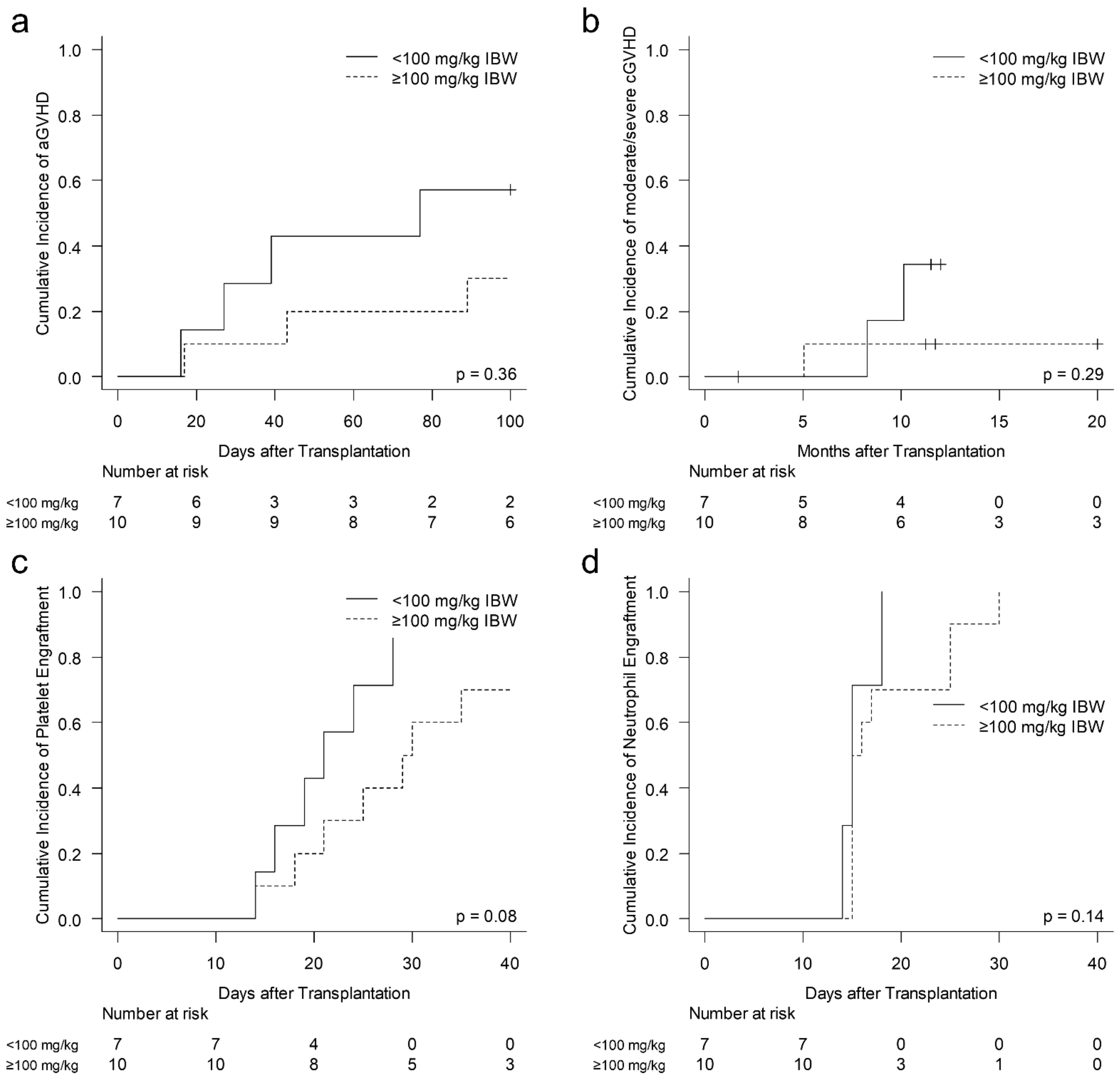

Supplementary Figure 2. Cumulative incidence of acute GVHD, moderate-to-severe chronic GVHD, and platelet and neutrophil engraftment according to the IBW-normalized PTCy dose in overweight/obese recipients.

(a–b) Cumulative incidence of (a) acute GVHD and (b) moderate-to-severe chronic GVHD, stratified by the IBW-normalized PTCy dose (<100 mg/kg IBW vs ≥100 mg/kg IBW). Cumulative incidence was estimated using the cumulative incidence function, treating relapse, subsequent transplantation, and death as competing events; groups were compared using Gray's test. Tick marks indicate censoring; numbers at risk are shown.

(c–d) Cumulative incidence of (c) platelet and (d) neutrophil engraftment, stratified by the IBW-normalized total PTCy dose (<100 mg/kg IBW vs ≥100 mg/kg IBW). The median times to platelet engraftment were 21 and 29 days, respectively, and the median times to neutrophil engraftment were 15 and 15 days, respectively. Cumulative incidence was estimated using the cumulative incidence function, treating relapse and death without engraftment as competing events; groups were compared using Gray's test. Tick marks indicate censoring; numbers at risk are shown.

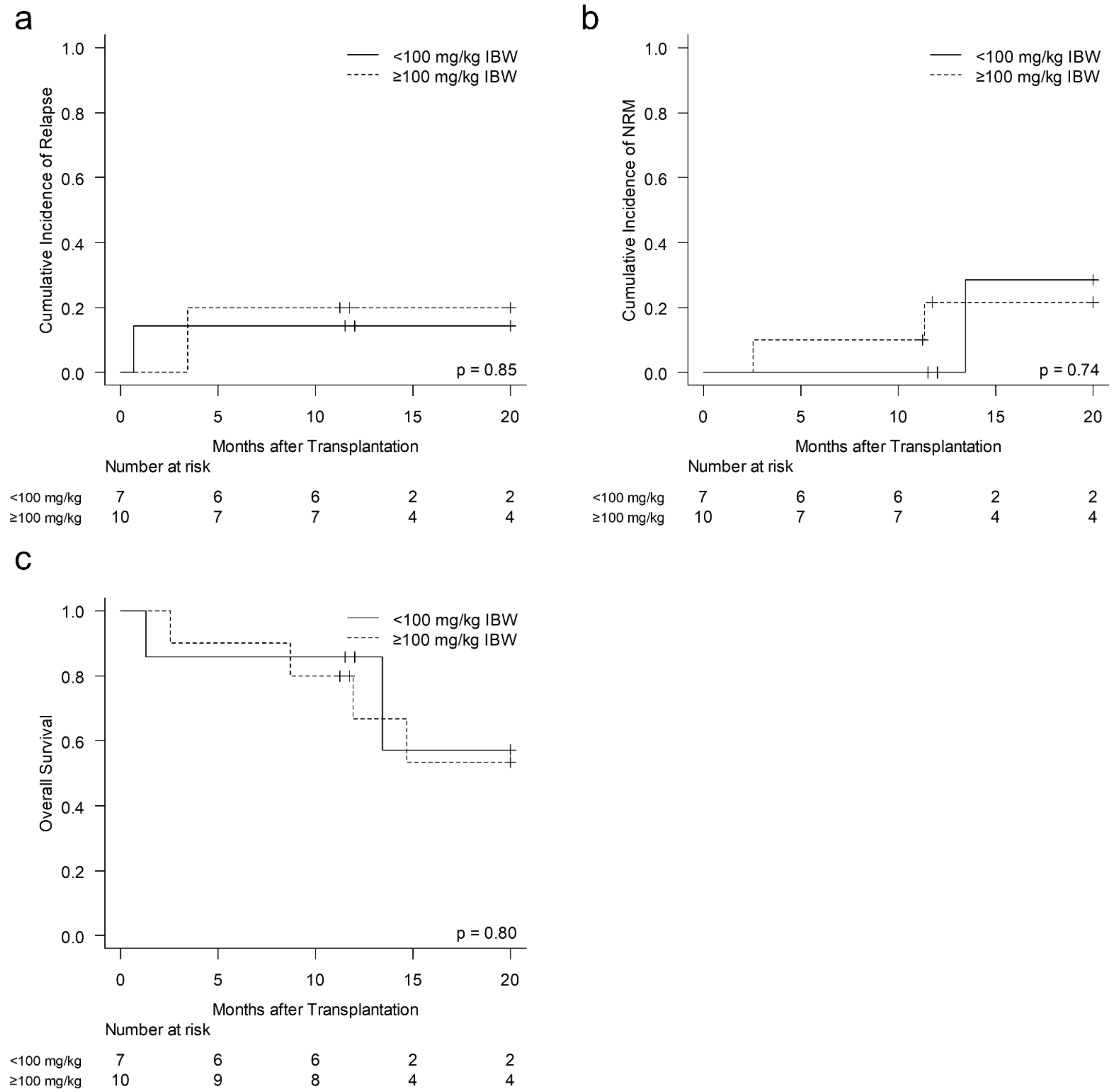

Supplementary Figure 3. Cumulative incidence of relapse and non-relapse mortality, and overall survival according to the IBW-normalized PTCy dose in overweight/obese recipients.

(a–b) Cumulative incidence of (a) relapse and (b) non-relapse mortality, stratified by the IBW-normalized total PTCy dose (<100 mg/kg IBW vs ≥100 mg/kg IBW). Cumulative incidence was estimated using the cumulative incidence function, treating death as the competing event for relapse and relapse as the competing event for non-relapse mortality; groups were compared using Gray's test. Tick marks indicate censoring; numbers at risk are shown.

(c) Overall survival stratified by the IBW-normalized total post-transplant cyclophosphamide (PTCy) dose (<100 mg/kg IBW vs ≥100 mg/kg IBW). Overall survival was estimated using the Kaplan–Meier method and compared using the log-rank test. Numbers at risk are shown.

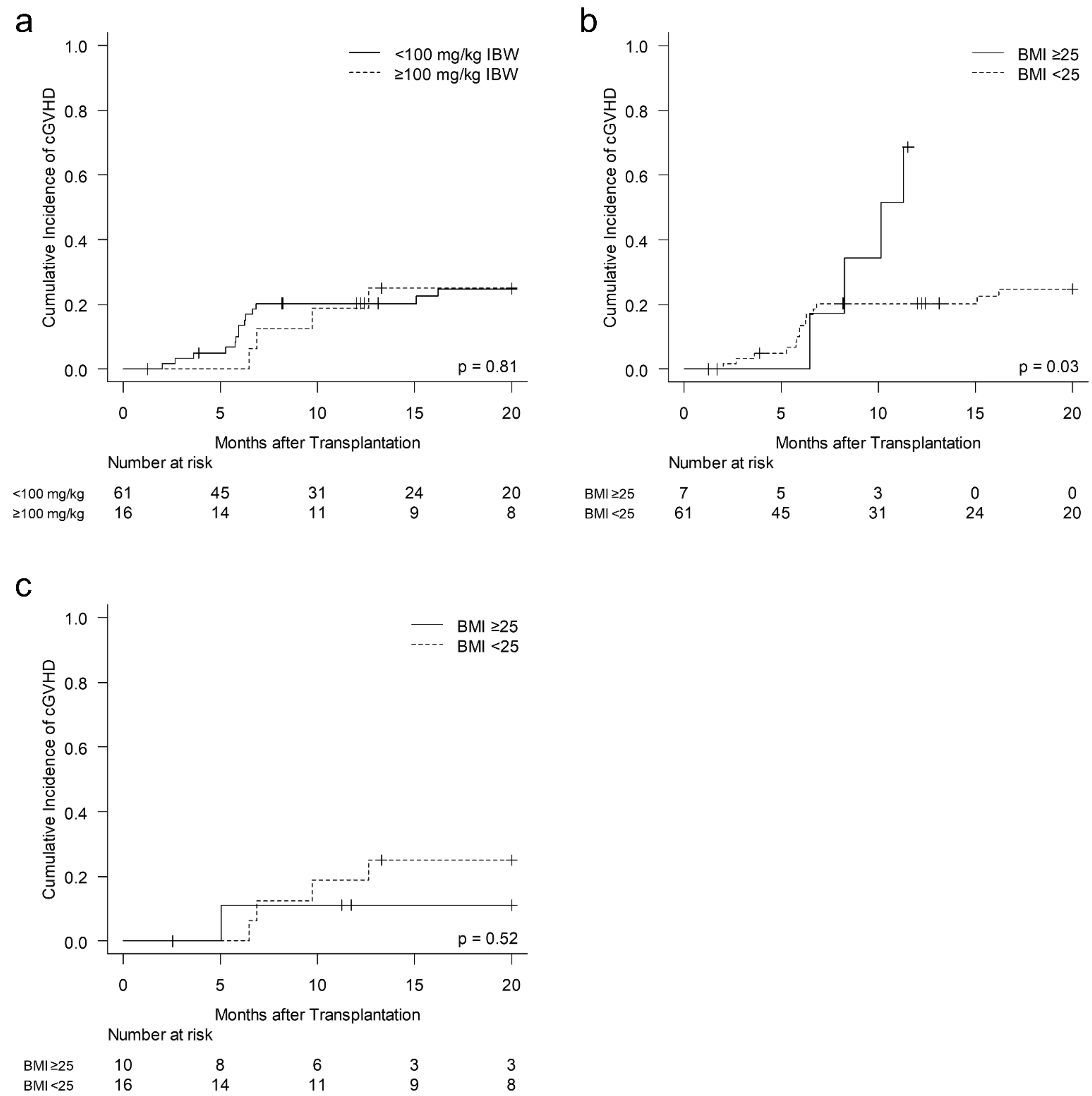

Supplementary Figure 4. Comparison of cumulative incidence of chronic GVHD between overweight/obese and non-overweight recipients.

(a) Among non-overweight recipients (BMI <25.0 kg/m²), cumulative incidence of chronic GVHD was compared between recipients who received <100 mg/kg IBW and those who received ≥100 mg/kg IBW total PTCy.

(b) Among recipients who received <100 mg/kg IBW total PTCy, cumulative incidence of chronic GVHD was compared between non-overweight recipients (BMI <25.0 kg/m²) and overweight/obese recipients (BMI ≥25.0 kg/m²).

(c) Among recipients who received ≥100 mg/kg IBW total PTCy, cumulative incidence of chronic GVHD was compared between non-overweight recipients and overweight/obese recipients.

For all panels, cumulative incidence was estimated using the cumulative incidence function, treating relapse, subsequent transplantation, and death as competing events; groups were compared using Gray's test. Tick marks indicate censoring; numbers at risk are shown below each plot.

Supplementary Methods

Definitions

Total body weight (TBW) was measured before the start of conditioning, and adjusted body weight (ABW25) was calculated as ideal body weight (IBW) + 0.25 × (TBW − IBW). Myeloablative and reduced-intensity conditioning were defined according to the criteria of the Center for International Blood and Marrow Transplant Research (CIBMTR) [12].

Acute and chronic GVHD were diagnosed and graded according to the modified Glucksberg criteria [13] and the National Institutes of Health consensus criteria [14], respectively. Neutrophil engraftment was defined as the first of 3 consecutive days on which the neutrophil count was ≥0.5 × 10^9/L. Platelet engraftment was defined as the first of 7 consecutive days on which the platelet count was ≥20 × 10^9/L without platelet transfusion support.

Cytomegalovirus (CMV) reactivation was defined as positive pp65 antigenemia or polymerase chain reaction (PCR) result during follow-up. CMV infection was defined as CMV reactivation with clinical manifestations and organ involvement attributable to CMV, requiring anti-CMV therapy. Hemorrhagic cystitis was diagnosed based on clinical findings. In patients diagnosed with hemorrhagic cystitis, urine BK virus (BKV) and

adenovirus (ADV) testing was performed. Cases with detectable BKV were classified as BKV-associated hemorrhagic cystitis. Cases negative for both BKV and ADV were classified as hemorrhagic cystitis without virus detection. Veno-occlusive disease/sinusoidal obstruction syndrome was diagnosed by the treating physician according to the 2016 or 2023 European Society for Blood and Marrow Transplantation (EBMT) criteria, as applicable [15, 16].

The cardiac events were assessed as arrhythmia, acute heart failure, left ventricular ejection fraction (LVEF) decline, and new-onset pericardial effusion. Arrhythmia was defined as a new-onset clinically significant arrhythmia documented by electrocardiography or telemetry and recorded in the medical record within 10 days after the first PTCy dose, excluding isolated premature atrial contractions and premature ventricular contractions. Continuous electrocardiographic monitoring was routinely performed during PTCy administration, and further cardiac monitoring was performed at the treating physician's discretion. Acute heart failure was evaluated within 100 days after transplantation and was defined as a diagnosis of acute heart failure made by the treating physician in consultation with a cardiologist. In our department, patients with suspected acute heart failure were routinely referred to a cardiologist for evaluation. LVEF decline was assessed among patients with available pre-transplant echocardiography and post-

transplant echocardiography performed within 100 days after transplantation and was defined as a decrease in left ventricular ejection fraction by ≥10 percentage points from baseline to a value <50%. LVEF was measured using Simpson's method. Pre-transplant echocardiography was performed in all patients, and post-transplant echocardiography was available in 13 of 17 patients who were overweight or obese. Pericardial effusion was assessed using computed tomography (CT) within 100 days after transplantation to allow sensitive detection of subclinical events. Pre-transplant CT was available in all patients, and 16 of 17 patients who were overweight or obese had no pericardial effusion at baseline. Among these patients, newly detected pericardial effusion on post-transplant CT was counted as an event. Post-transplant CT was performed in all patients.

Statistical analysis

Statistical analyses were performed using EZR version 1.65 [17]. Categorical and continuous variables were compared using Fisher's exact test and the Wilcoxon rank-sum test, respectively. Overall survival was estimated using the Kaplan-Meier method and compared using the log-rank test. Exploratory stratified analyses were conducted within clinically relevant subgroups to assess potential confounding factors. The evaluated subgroups are shown in Supplementary Table 2.

Supplementary References

12. Giralt S, Ballen K, Rizzo D, Bacigalupo A, Horowitz M, Pasquini M, *et al*. Reduced-intensity conditioning regimen workshop: defining the dose spectrum. Report of a workshop convened by the center for international blood and marrow transplant research. Biol Blood Marrow Transplant. 2009; 15:367–369.

13. Przepiorka D, Weisdorf D, Martin P, Klingemann HG, Beatty P, Hows J, *et al.* 1994 Consensus conference on acute GVHD grading. Bone Marrow Transplant. 1995; 15:825–828.

14. Filipovich AH, Weisdorf D, Pavletic S, Socie G, Wingard JR, Lee SJ, *et al.* National Institutes of Health consensus development project on criteria for clinical trials in chronic graft-versus-host disease: I. Diagnosis and staging working group report. Biol Blood Marrow Transplant. 2005; 11:945–956.

15. Mohty M, Malard F, Abecassis M, Aerts E, Alaskar AS, Aljurf M, *et al.* Revised diagnosis and severity criteria for sinusoidal obstruction syndrome/veno-occlusive disease in adult patients: a new classification from the European Society for Blood and Marrow Transplantation. Bone Marrow Transplant. 2016; 51:906–912.

16. Mohty M, Malard F, Alaskar AS, Aljurf M, Arat M, Bader P, *et al.* Diagnosis and severity criteria for sinusoidal obstruction syndrome/veno-occlusive disease in adult

patients: a refined classification from the European society for blood and marrow transplantation (EBMT). Bone Marrow Transplant. 2023; 58:749–754.

17. Kanda Y. Investigation of the freely available easy-to-use software "EZR" for medical statistics. Bone Marrow Transplant. 2013; 48:452–458.
